# A Clinical Model for the Prediction of Acute Exacerbation Risk in Patients with Idiopathic Pulmonary Fibrosis

**DOI:** 10.1155/2020/8848919

**Published:** 2020-12-07

**Authors:** Qi Wu, Yong Xu, Ke-jia Zhang, Shi-min Jiang, Yao Zhou, Ying Zhao

**Affiliations:** ^1^Department of Physiology, Xuzhou Medical University, Xuzhou 221009, China; ^2^Affiliated Hospital of Nanjing University of Chinese Medicine, Nanjing 210029, China; ^3^Department of Pathophysiology, Xuzhou Medical University, Xuzhou 221009, China; ^4^Laboratory of Clinical and Experimental Pathology, Xuzhou Medical University, Xuzhou 221009, China; ^5^Xuzhou Traditional Chinese Medicine Hospital Affiliated to Nanjing University of Chinese Medicine, Xuzhou 221009, China

## Abstract

**Objective:**

To develop and validate a risk assessment model for the prediction of the acute exacerbation of idiopathic pulmonary fibrosis (AE-IPF) in patients with idiopathic pulmonary fibrosis (IPF).

**Methods:**

We enrolled a total of 110 patients with IPF, hospitalized or treated as outpatients at Xuzhou Traditional Chinese Medicine Hospital Affiliated to Nanjing University of Chinese Medicine from July 2012 to July 2020. Of these, 78 and 32 patients were randomly assigned to training and test groups, respectively. The risk factors for AE-IPF were analyzed using logistic regression analysis, and a nomographic model was constructed. The accuracy, degree of calibration, and clinical usefulness of the model were assessed with the consistency index (C-index), calibration diagram, and decision curve analysis (DCA). Finally, the stability of the model was tested using internal validation.

**Results:**

The results of logistic regression analysis showed that a history of occupational exposure, diabetes mellitus (DM), essential hypertension (EH), and diffusion capacity for carbon monoxide (DLCO)% predicted were independent risk factors for AE-IPF prediction. The nomographic model was constructed based on these independent risk factors, and the C-index was 0.80. The C-index for the internal validation was 0.75, suggesting that the model had good accuracy. The decision curve indicated that for a threshold value of 0.04–0.66, greater clinical benefit was obtained with the AE-IPF risk prediction model.

**Conclusion:**

A customized AE-IPF prediction model based on a history of occupational exposure, DM, EH, and DLCO% predicted provided a reference for the clinical prediction of AE-IPF.

## 1. Introduction

Idiopathic pulmonary fibrosis (IPF) is a chronic progressive interstitial lung disease characterized by severe pulmonary tissue fibrosis without a known pathogenesis; the mean survival time after diagnosis is 2–3 years[1]. The occurrence and development of IPF is related to epithelial cell injury, the proliferation of fibroblasts, inflammatory response, and extracellular matrix deposition [2, 3]. The pathologic feature of IPF is usual interstitial pneumonia (UIP). Unexpected respiratory failure, known clinically as the acute exacerbation of IPF (AE-IPF), usually occurs in a proportion of patients with IPF [4–6].

AE-IPF was first reported in 1984. Yoshimura et al. briefly reported the symptoms and mortality rate of AE-IPF and identified the efficacy of a large dosage of glucocorticoids [7]. It was not until 2002 that the terminology of AE-IPF was officially acknowledged in the clinical consensus of interstitial lung diseases.

Although multiple retrospective studies have reported on the incidence and mortality rate of AE-IPF, a consensus has not been reached. This can be attributed to the different study designs, inconsistent standards for disease diagnosis, few cases, and loss of patients to follow-up [8]. In a retrospective study of 461 IPF cases, AE-IPF was identified as the major cause for the deterioration of IPF, accounting for 55.2% of the cases. In another study, the incidence of AE-IPF was 14.2% and 20.7% at 1 and 3 years after IPF, respectively [9]. The median overall survival of AE-IPF patients was only 2.2 months; of these patients, 50% died during hospitalization and 40% died in the ICU (mortality rate > 90%) [9].

Currently, there are no effective therapies for the treatment of AE-IPF. Internationally acknowledged suggestions include supportive nursing and glucocorticoid treatment which aim to alleviate symptoms and rectify hyperemia; however, the effects of these strategies are limited [10]. Therefore, it is pertinent to construct a risk assessment model to evaluate the risk factors for AE-IPF in patients with IPF. A wealth of literature has documented that inheritable factors, air pollution, seasonal factors, partial deterioration in lung function (assessed via forced vital capacity (FVC), diffusion capacity for carbon monoxide (DLCO), 6 min walking test, and St. George's respiratory questionnaire), pulmonary arterial hypertension, and coronary artery disease may all increase the risk for AE-IPF [11]. However, a model for the prediction of AE-IPF is lacking. Consequently, in this study, multivariate logistic regression analysis was used to identify high-risk factors for AE-IPF, in order to analyze the degree of risk, construct a simple prediction model, and provide an effective tool for the prediction of AE-IPF.

## 2. Patients and Methods

### 2.1. Patients

Patients with IPF who were hospitalized in or visited Xuzhou Traditional Chinese Medicine Hospital Affiliated to Nanjing University of Chinese Medicine from July 2012 to July 2020 were enrolled in the present study. In total, 110 patients were included; 78 patients were selected for the construction of the risk analysis model, and the remaining 32 patients were used to test the model. For patients with multiple hospitalizations or visits, the data from the first hospitalization or visit were adopted. The patients provided written informed consent for inclusion in this study, and the study was approved by the Ethics Commission of Xuzhou Hospital of Traditional Chinese Medicine (ID: XZTCM2018LSY-013).

### 2.2. Diagnostic Criteria

Patients with IPF were confirmed based on clinical features, imaging data, and medical history according to the diagnostic criteria for IPF (2011) which included the following: (1) interstitial pneumonia with known etiology (family or occupational exposure, desmosis, and drug dependency) and (2) HRCT-indicated UIP or suspected UIP symptoms [12]. The diagnostic criteria for AE-IPF were as follows: (1) past or present IPF, (2) development or occurrence of deteriorative acute dyspnea within 1 month, (3) newly formed bilateral frosted glass shadow or consolidation shadow at the original reticular shadow or beehive-like shadow UIP via thoracic HRCT, and (4) deterioration of dyspnea that could not be explained by heart failure or liquid overload [13].

### 2.3. Data Collection

Clinical data were obtained from the medical history of the patients during hospitalization and the follow-up visits. The data included the following: (1) patient characteristics, such as age, sex, occupational history, and history of smoking; (2) complications, such as diabetes mellitus (DM), essential hypertension (EH), coronary heart disease (CHD), cerebral infarction (CI), hypothyroidism, gastroesophageal reflux disease (GERD), and obstructive sleep apnea-hypopnea syndrome (OSAHS); (3) lung function test indices, such as Forced Vital Capacity (FVC)% predicted and diffusing capacity for carbon monoxide (DLCO)% predicted; and (4) medical treatment, including *N*-acetylcysteine (NAC), pirfenidone (PFD), glucocorticoid (GC), and acid-inhibitory drugs.

### 2.4. Statistical Analysis

All clinical data are shown as count (%) and were analyzed with R version 3.6.3 (R Foundation for Statistical Computing, Vienna, Austria). The Caret package in R was used to randomly assign the 110 patients to the training and test groups in a ratio of 7 : 3 (78 cases and 32 cases, respectively). Information on the training group was subjected to multivariate logistic regression with the odds ratio, 95% confidence interval, and*p*value as characteristics; a *p* value of <0.05 was considered statistically significant. Factors describing statistical significance were used to construct the nomogram using the “rms” program to create a prediction model. To test the accuracy of the prediction model, we used the “rms” program to calculate the consistency index (C-index). The degree of calibration was evaluated by constructing a calibration curve; the closer the calibration curve of the prediction model to the standard curve, the better the consistency of the prediction model. The area under curve (AUC) value of the model was calculated to predict the degree of discrimination of the prediction model, with a higher AUC value representing a higher degree of discrimination. By quantifying the net benefits of the probability of different threshold values in the array, decision curve analysis (DCA) was used to determine the clinical usefulness of the nomogram. Finally, an internal validation method was used to test the stability of the prediction model; the “rms” program package was used to calculate the C-index and AUC value for the 32 patients with IPF in the test group.

## 3. Results

### 3.1. Patient Characteristics

In total, 110 patients with IPF, treated as inpatients or outpatients between July 2012 and July 2020, were enrolled in the present study. In the training group (78 cases), there were 16 patients with AE-IPF (20.51%), while in the test group (32 cases), there were four patients with AE-IPF (12.50%). All the patients' clinical data, including demographic and clinical characteristics, complications, lung function, and medication, are presented in Table 1.

### 3.2. Analysis of Risk Factors for AE-IPF

The clinical data of the training group were entered into the multivariate logistic regression model to obtain the coefficients of the corresponding characteristic variables. Among the variables, a history of occupational exposure (*p* < 0.01), DM (*p* < 0.01), EH (*p* < 0.01), and DLCO%-predicted lung function (*p* < 0.05) were identified as the risk factors for AE-IPF (Table 2).

### 3.3. Nomography of AE-IPF Risk

**Figure 1:**
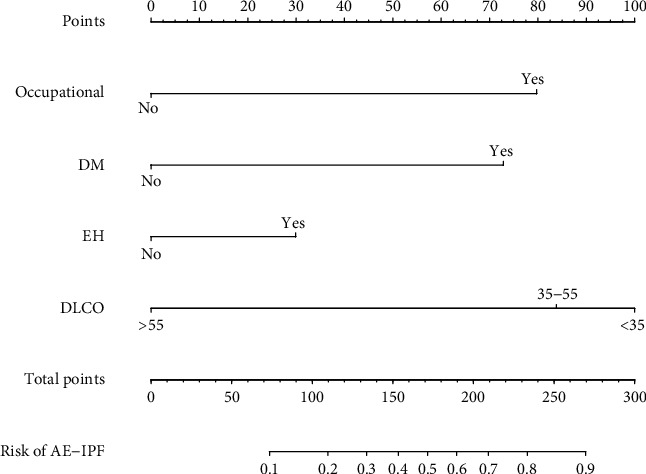
Nomogram to predict the incidence of AE-IPF. The nomogram was constructed with occupational exposure, DM, EH, and DLCO% predicted. Abbreviations: DM: diabetes mellitus; EH: essential hypertension; DLCO: diffusion capacity for carbon monoxide.

Based on the results of the logistic regression, we determined the predictive factors for AE-IPF. To facilitate the estimation of the risk for AE-IPF, we screened the four characteristic variables and then constructed a nomographic risk prediction model. Each variable was scored on a scale, and the range of the total score was 0–220. The total score on the AE-IPF risk axis represents the probability of AE-IPF. The higher the score, the higher the risk that a patient with IPF will develop AE-IPF (Figure 1).

### 3.4. Assessment of the Degree of Calibration for the Risk Prediction Model of AE-IPF

**Figure 2:**
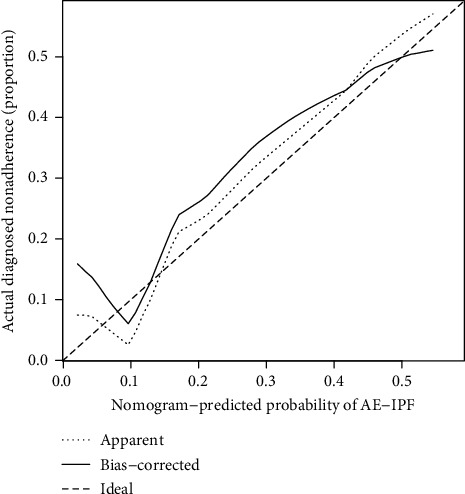
Calibration curve for the risk prediction model of AE-IPF.

We constructed calibration curves to facilitate the assessment of the degree of calibration of the risk prediction model of AE-IPF. As shown in Figure 2, the *x*-axis represents the predicted risk for AE-IPF, and the *y*-axis represents the realization of AE-IPF. The bidiagonal dotted line represents the prediction of an ideal model, and the solid line represents the realized prediction capacity. The closer the dotted line is to the bidiagonal line, the greater the prediction capacity. The results of the present study showed a preferable consistency between the nomographic model and the ideal model.

### 3.5. Assessment of the Accuracy of AE-IPF Risk Prediction

**Figure 3:**
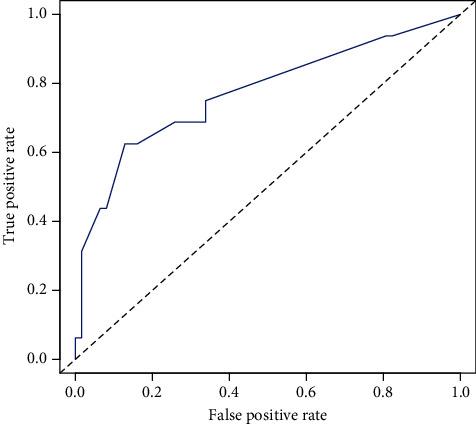
ROC curve corresponding to the risk prediction model of AE-IPF. The AUC was 0.77.

To test the accuracy of the model, we first calculated the C-index. An ROC curve indicates that the predictions are close to random guesses. Meanwhile, when an ROC curve is plotted, we can use AUC to measure the performance of the predictor. We then calculated the AUC value of the model and constructed an ROC curve [12]. Based on the calculation, the C-index was 0.80, suggesting good accuracy of the model. As shown in Figure 3, the AUC of the ROC curve was 0.77, suggesting good accuracy of the AE-IPF risk prediction model.

### 3.6. Internal Validation of the Model for AE-IPF Risk Prediction

**Figure 4:**
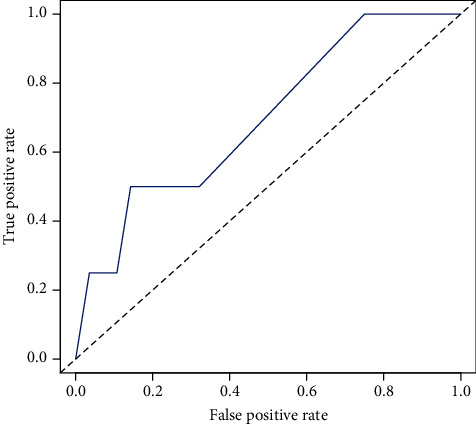
ROC curve corresponding to the test group. The AUC was 0.70.

To further evaluate the stability of the AE-IPF risk prediction model, we calculated the C-index and AUC values of the 32 patients with IPF in the test group using the internal validation group. The C-index value was 0.75, and after constructing the ROC curve to calculate the AUC value for further validation, the AUC value was 0.70 (Figure 4). Therefore, both the C-index and AUC confirmed the good stability of the AE-IPF risk prediction model.

### 3.7. Clinical Net Benefit

**Figure 5:**
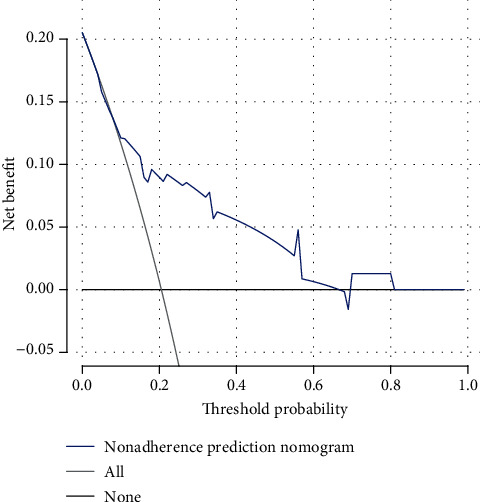
DCA for the risk prediction model of AE-IPF. The decision curve showed that, at threshold values of 0.04–0.66, using this nomogram to predict the risk of AE-IPF added more benefit.

DCA was used to evaluate whether the prediction model could improve clinical decision-making. The decision curve of the prediction model is shown in Figure 5. The *y*-axis indicates the net benefit, and the *x*-axis represents the probability of the threshold value. The black solid line represents no intervention, at which the net benefit is zero. The black dotted line represents the intervention, and the net benefit is an oblique line with a negative slope. The blue solid line represents the realized profits of the AE-IPF risk prediction model. The decision curve showed that the greatest clinical benefit will be obtained from the AE-IPF risk prediction model at threshold values of 0.04–0.66.

## 4. Discussion

At present, the cause of AE-IPF is unknown. Recurrent local outbreaks of alveolar damage may lead to the progression of IPF [14]. For some patients, recurrent outbreaks of alveolar damage will inflict damage and worsen their condition, eventually leading to acute exacerbation [15]. In addition, it has been suggested that infectious factors and some direct lung injury can also induce the pathophysiological processes of AE-IPF [16]. AE-IPF can occur at any stage during the process of IPF, and AE-IPF may even occur during the relatively stable stage of IPF [17]. Therefore, it is important to predict the occurrence of AE-IPF. However, a reliable model for the prediction of AE-IPF is lacking. In the present study, a model was created to predict the risk of AE-IPF and to provide a reference for the clinical prediction of AE-IPF.

Based on the prediction model, occupational exposure is a major risk factor for AE-IPF. Therefore, workers engaged in ore mining, construction work, inorganic dust production, and chemical processing and manufacturing, and those exposed to organic dust production, are at high risk. Owing to a lack of research, the association between occupational exposure and a strong predisposition toward AE-IPF is unknown. It is likely that when productive chemicals with systemic toxicity enter the respiratory tract in the form of aerosols or gas, they readily enter the blood flow through the vascular bed in the lung, leading to respiratory system damage, systemic poisoning, and AE-IPF.

In addition to IPF itself, some patients with IPF also have other complications, which may also induce AE-IPF [18]. According to the prediction model, DM and EH are high-risk factors for AE-IPF. It is suggested that DM is an independent factor for the occurrence of IPF [19]. Patients with DM usually experience aberrant lung function, mainly characterized by impairments in lung ventilation, malfunctions in lung diffusion, and abnormalities of bronchomuscular tension and respiratory muscle function [20]. It has also been reported that the malfunction of pulmonary ventilation in DM patients can become compounded over the course of the disease [21]. However, it is unclear why DM leads to IPF exacerbation. It is possible that the final metabolites of DM cause airway endothelial cell injury and abnormalities in the airway defense mechanism, which eventually leads to the progression of IPF. Regarding EH, the association between EH and AE-IPF has received less attention. However, according to the prediction model, EH is another risk factor for the occurrence of AE-IPF. This should be proven by a large-sized clinical trial in the future. Therefore, the treatment of IPF requires a systematic approach, including time discrimination and the treatment of DM, EH, and other complications, in order to optimize treatment efficiency.

Lung function measurement is an important method for IPF diagnosis and for assessing the state of illness [22]. According to the consensus for treatment and diagnosis of IPF issued by ATS/ERS in 2011, a ≥10% reduction in FVC compared with the absolute value at baseline and a ≥15% reduction in DLCO% predicted suggest a good prognosis for IPF [23]. This highlights the significance of FVC and DLCO% predicted in diagnosing IPF and assessing the state of illness in IPF. Based on the prediction model, FVC is not a notable risk factor for AE-IPF following logistic regression analysis, whereas DLCO% predicted was an important predictor of AE-IPF. It is suggested in the 2011 guidelines that the lung function of patients with IPF should be examined every 3–6 months. Pulse oximetry saturation during rest and walking in patients with IPF should also be measured to determine the necessity of oxygen treatment.

To evaluate the accuracy of the prediction model, we first calculated the C-index and AUC values. The C-index was 0.80, and the AUC value was 0.77, suggesting that the AE-IPF risk prediction model has an acceptable accuracy. To further examine the stability of the prediction model, we used an internal validation method, by evaluating the clinical data of the 32 patients with IPF in the test group, and then calculated the C-index and AUC values. The C-index and AUC values were 0.75 and 0.70, respectively. The results of the internal validation thus suggest acceptable stability of the AE-IPF risk prediction model.

## 5. Conclusion

A history of occupational exposure, DM, EH, and DLCO% predicted were risk factors for AE-IPF as determined by the logistic regression analysis, and the AE-IPF risk prediction model was successfully constructed by creating a nomogram. The nomogram could clearly and intuitively predict the probability of IPF patients' progress to AE-IPF, thus supplying a reference for clinical progression. However, the current study was a single-center retrospective study with a limited sample size. Therefore, the results of this study should be confirmed in a prospective multicenter, large-scale study. In addition, hierarchical studies of patients of different ages or with different illness severities should also be performed.

## Figures and Tables

**Table 1:** Patient characteristics of the training and test groups.

Characteristic	Training group (*n* = 78)	Verification group (*n* = 32)
AE-IPF	16 (20.51)	4 (12.50)
Demographic and clinical characteristics		
Gender		
Male	48 (61.54)	18 (56.25)
Female	30 (38.46)	14 (43.75)
Age		
≤60	9 (11.54)	4 (12.50)
61-65	11 (14.10)	2 (6.250)
>65	58 (74.36)	26 (81.25)
Occupational	8 (10.26)	3 (9.38)
Smoking	16 (20.51)	4 (12.50)
Comorbidities		
DM	17 (21.79)	3 (9.38)
EH	7 (8.97)	6 (18.75)
CHD	10 (12.82)	4 (12.50)
CI	11 (14.10)	5 (15.63)
GERD	11 (14.10)	6 (18.75)
Hypothyroidism	4 (5.13)	1 (3.13)
OSAHS	3 (3.85)	0
Lung function		
FVC% predicted		
>75	16 (20.51)	4 (12.50)
50-75	47 (60.26)	24 (75.00)
<50	15 (19.23)	4 (12.50)
DLco% predicted		
>55	19 (24.36)	6 (18.75)
35-55	41 (52.56)	20 (62.50)
<35	18 (23.08)	6 (18.75)
Drug therapy		
Acetylcysteine	16 (20.51)	3 (9.38)
Pirfenidone	5 (6.41)	0
Glucocorticoid	12 (15.38)	4 (12.50)
Acid-inhibitory drugs	5 (6.41)	2 (6.25)

Abbreviations: AE-IPF: acute exacerbation of idiopathic pulmonary fibrosis; DM: diabetes mellitus; EH: essential hypertension; CHD: coronary heart disease; CI: cerebral infarction; GERD: gastroesophageal reflux disease; OSAHS: obstructive sleep apnea-hypopnea syndrome; FVC: forced vital capacity; DLCO: diffusion capacity for carbon monoxide; NAC: *N*-acetylcysteine; PFD: pirfenidone; GC: glucocorticoid.

**Table 2:** Final regression model for the primary outcome.

Intercept	*β*	Odds ratio (95% CI)	*p* value
Intercept	-5.94	0.00 (0.00-0.23)	0.03
Occupational	5.59	267.90 (6.47-40395.40)	0.01
DM	3.76	42.76 (3.84-1227.58)	0.01
EH	-4.50	0.01 (0.00-0.27)	0.02
DLCO% predicted			
36-55	3.50	32.97 (1.84-2038.88)	0.04
<35	3.97	52.73 (2.22-3825.05)	0.03

Abbreviations: DM: diabetes mellitus; EH: essential hypertension; DLCO: diffusion capacity for carbon monoxide.

## Data Availability

The data used to support the finding of this study are available from the corresponding author upon request.
